# The hydrogenation of mandelonitrile over a Pd/C catalyst: towards a mechanistic understanding†

**DOI:** 10.1039/c9ra04618f

**Published:** 2019-08-21

**Authors:** Mairi I. McAllister, Cédric Boulho, Liam McMillan, Lauren F. Gilpin, Colin Brennan, David Lennon

**Affiliations:** School of Chemistry, University of Glasgow Joseph Black Building Glasgow G12 8QQ UK David.Lennon@glasgow.ac.uk +44(0)-141-330-4372; Syngenta, Jeallot's Hill International Research Centre Bracknell Berkshire RG42 6EY UK

## Abstract

A carbon supported Pd catalyst is used in the liquid phase hydrogenation of the aromatic cyanohydrin mandelonitrile (C_6_H_5_CH(OH)CH_2_CN) to afford the primary amine phenethylamine (C_6_H_5_CH_2_CH_2_NH_2_). Employing a batch reactor, the desired primary amine is produced in 87% selectivity at reaction completion. Detection of the by-product 2-amino-1-phenylethanol (C_6_H_5_CH(OH)CH_2_NH_2_) accounts for the remaining 13% and closes the mass balance. The reaction mechanism is investigated, with a role for both hydrogenation and hydrogenolysis processes established.

## Abstract 2

A carbon supported Pd catalyst is used in the liquid phase hydrogenation of the aromatic cyanohydrin mandelonitrile (C_6_H_5_CH(OH)CH_2_CN) to afford the primary amine phenethylamine (C_6_H_5_CH_2_CH_2_NH_2_).

## Graphical abstract

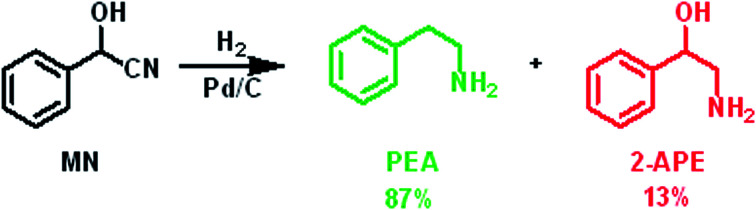

## 1. Introduction

Primary amines are recognised as an industrially valuable species utilised in the production of many products, including plastics, pharmaceuticals and agrichemicals.^1,2^ Phenethylamine is representative of such a species, and whilst various synthetic methods, for example the alkylation of ammonia and reductive amination, are currently available to afford such compounds, it is the heterogeneously catalysed hydrogenation of nitriles which is frequently employed for this purpose.^3–5^

**Scheme 1:**
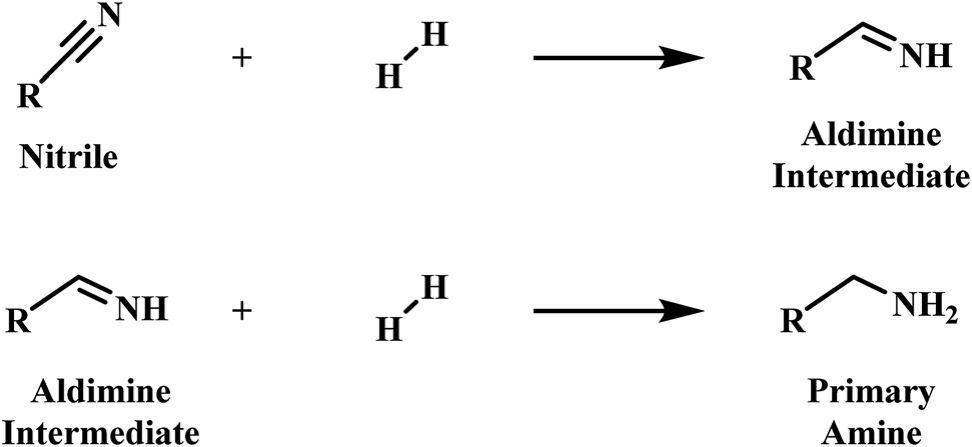
Two-step nitrile hydrogenation to the primary amine proposed by Sabatier *et al.*^6^

Early mechanistic work on the hydrogenation of nitriles was undertaken by Sabatier and Senderens.^6^ Here it was proposed that the nitrile is hydrogenated to the primary amine in a two-step process *via* a primary aldimine intermediate (Scheme 1).^6^ The highly reactive nature of the intermediate species in the hydrogenation of nitriles, however, meant that identification of the intermediate species in the hydrogenation of nitriles initially proved problematic.^7,8^ Nonetheless, the presence of imines and enamines as intermediate species is now firmly established.^9–11^

Braun *et al.* undertook further development of the mechanism in 1923,^12^ It was suggested that, as a consequence of their reactivity, side reactions, involving the aldimine intermediates, occurring alongside the main hydrogenation reaction were inevitable.^8,13^ The coincident formation of these undesirable by-products can be problematic in a reaction system if they are retained on the catalyst surface, as they can act as a poison and cause deactivation.^14,15^

Another pathway, commonly seen alongside the hydrogenation of the nitrile species, is the hydrogenolysis of the primary amine product.^16,17^ Historically, this heteroatom cleavage has not been a predominant feature of the heterogeneous catalysis literature, with the phenomenon typically only occurring under harsh reaction conditions.^17,18^ Despite this, production of the hydrogenolysis product under mild conditions has been reported, for example, by Maschmeyer *et al.*,^17^ through the study of benzonitrile over a Pd/C catalyst. Whilst there are several instances where hydrogenolytic removal of a functional group is undesirable, such as the hydrogenolysis of benzylamine to yield toluene,^19,20^ there are occasions where no hydrogenolysis of the amine functionality is observed.^21^ Adding a further degree of complication, there are instances where the selective hydrogenolysis of one functional group in the presence of another is required.^21^ The occurrence or absence of a hydrogenolysis step has previously been rationalised, primarily by work undertaken by Maschmeyer *et al.*,^17^ which suggested that it is the positioning of the heteroatom with respect to the aromatic group which dictates the viability of the process. Recent work by McAllister and co-workers regarding the hydrogenation of 4-hydroxybenzyl cyanide supports this assertion.^21^

Whilst aliphatic nitriles and, more recently, simple aromatic nitriles such as benzonitrile, have been extensively studied, chemical systems of greater complexity have not been examined in as much detail.^2,3,22,23^ Cyanohydrins are organic compounds defined by the presence of both a nitrile group and a hydroxyl group on the same carbon atom.^24^ Found commonly in nature, the inherent chirality of these species offers significant synthetic potential,^25–28^ and, as such, their synthesis is recurrently reported.^29–32^ Interestingly, however, the literature available on the hydrogenation of these compounds is rather scarce, featuring significantly less frequently than non-substituted aromatic or aliphatic nitriles.^26,33,34^ Indeed, despite the industrial importance of this class of reaction, and to the best knowledge of the authors, there are no detailed accounts of the mechanism for an industrially scalable heterogeneously catalysed route from cyanohydrin to primary amine.

Historically, it has been skeletal metal catalysts such as RANEY® Ni and Co which have shown proclivity for the selective hydrogenation of nitriles.^11,13,35^ Nevertheless, catalysts of this nature are pyrophoric causing handling difficulties. Further, the high hydrogenation activity of RANEY® metal catalysts becomes problematic when more complex molecules, with additional functionality requiring preservation, are considered.^36^ Consequently, more recently, it has been the milder, supported Pd catalysts which have come to dominate the literature.^2,7,16,36,37^

Against this background, and in a move towards the consideration of more complex nitrile species, mandelonitrile (C_6_H_5_C(OH)CN) is selected as an ideal candidate to probe the nature of aromatic cyanohydrin hydrogenations over Pd/C. With direct application in an industrial agrichemical production chain (unspecified), the use of a carbon supported Pd catalyst has thus been selected for the conservation of the valuable amine functionality of the desired product. The route to the target primary amine phenethylamine (C_6_H_5_CH_2_CH_2_NH_2_) is found to exhibit a significant degree of complexity. A role for imine and enamine species, undetectable in the liquid phase and thus assumed to be adsorbed species, is evoked as a result of kinetic considerations, allowing the development of a global reaction scheme for the transformation of mandelonitrile to phenethylamine.

## 2. Experimental

### 2.1 Materials

Selected as a representative material suitable for use as a generic fine chemicals hydrogenation catalyst, a commercial grade 5% Pd/C catalyst (Sigma Aldrich, code number: 205680) was exclusively used. Characterisation of this catalyst has previously been reported.^19^ Briefly, the percentage metal loading was determined by atomic absorption spectroscopy (PerkinElmer AAnalyst 100 Atomic Absorption Spectrometer at 244.8 nm) and found to be 3.66%. Additionally, a complementary measurement conducted using Inductively Coupled Plasma-Optical Emission Spectrometry (Agilent 5100 calibrated to a range of known-concentration (ppm) solutions of Pd) revealed a percentage metal loading of 4.12%. Pulsed carbon monoxide chemisorption measurements were carried out in a quartz ‘u-tube’ reactor. A known volume of CO was then pulsed over the catalyst with saturation points measured by gas chromatography (Thermo Finnigan Ultra GC fitted with a thermal conductivity detector). A maximum CO capacity of 9.24 × 10^−5^ mol CO g_(catalyst)_^−1^ was obtained. Assuming a CO : Pd(s) ratio of 1 : 2,^38^ this equates to 1.11 × 10^20^ Pd(s) atoms per g_(catalyst)_, which corresponds to a Pd dispersion of 39% and a mean particle size of 2.8 nm. In agreement with CO chemisorption data TEM analysis (Tecnai G2T20 STWIN fitted with a tungsten filament at 200 kV) reveals a narrow particle size distribution with the majority of particles ranging from 2–3 nm in diameter. Nitrogen physisorption (Quantachrome Evo with prior degassing-Quantachrome FloVac) revealed a type IV isotherm,^39^ with type H4 hysteresis.^40^ and a specific surface area of 775 ± 19 m^2^ g^−1^. Further catalyst characterisation details may be found in the ESI (Fig. S1–S9).†

A variety of substrates, racemic mandelonitrile (Tokyo Chemical Industry, >97% purity), 2-amino-1-phenylethanol (Sigma Aldrich, 98% purity), 2-aminoacetophenone hydrochloride (Alfa Aesar, 97% purity) and sulphuric acid (Fischer Scientific, nominally 95–98% H_2_SO_4_) have been used, as received, and without further purification. In all instances methanol (Riedel-de Haën, 99.9% purity) was utilised as the reaction solvent.

An automated gas flow controller (BPC 1202) allowed delivery of inert (N_2_, BOC, 99.9% purity) and active (H_2_, BOC, ≥99.8% purity) gases to be delivered to the reactor *via* a gas reservoir. Helium gas (BOC, 99.9% purity) was also employed for reagent degassing.

### 2.2 Hydrogenation reaction procedure

The hydrogenation reactions were carried out in a 500 mL stirred autoclave (Büchi Glas Uster). The reactor was first charged with catalyst (300 mg and solvent (310 mL methanol)) prior to being purged with inert gas (N_2_). The catalyst/solvent mixture was heated and stirred at 300 rpm under hydrogen for one hour in order to reduce the catalyst. The reaction vessel was heated by silicon oil passed around the reactor *via* a heating circulator (Julabo, F25). In a separate vessel, the substrate (11 mmol mandelonitrile, 11 mmol 2-amino-1-phenylethanol, or 2.75 mmol 2-aminoacetophenone) was combined with two molar equivalents of sulphuric acid, dissolved in 40 mL reaction solvent and degassed under helium.

Whilst an acid additive proved to be essential for this reaction (Section 3.2 and Fig. S10†), it should be noted that under neutral or basic conditions there is the potential production of hydrogen cyanide as a degradation product of mandelonitrile (Scheme S1†). As such, the reaction was carried out in a vented cabinet that provided secondary containment. Moreover, under the hydrogenation conditions presented here, it is chemically likely that any hydrogen cyanide produced will be hydrogenated to methylamine (Scheme S1†).^41^

Once the reaction vessel reached 40 °C, measured within the reactor by a thermocouple, substrate addition was made, the system was sealed and the hydrogen pressure elevated to 6 barg. When the reaction pressure was attained, agitation was commenced at 1050 rpm; this represents an agitation speed on the plateau region of a rate of mandelonitrile consumption *versus* agitation plot, thus indicating the reaction to be under kinetic control.^42^ The reaction start time occurs once the pressure of the system stabilises at the chosen value. The reaction typically commences during the short hydrogen pressure charging process; hydrogen up-take measurements cannot be reliably monitored during this procedure. However, once the reactor pressure is attained, hydrogen up-take measurements can be used to determine relative hydrogen consumption as a function of the reaction coordinate.

### 2.3 Analysis: high performance liquid chromatography

Liquid samples were collected periodically and filtered, using a 0.22 μm syringe filter (cellulose acetate, Chromatography Direct), to remove any catalyst residue. All samples, contained in a sealed vial, were subsequently quenched in ice until it was timely for their analysis by high performance liquid chromatography (HPLC). This procedure ensured the absence of unwanted homogeneous chemistry (Fig. S11†). HPLC analyses were obtained using a Hewlett Packard 1100 Series HPLC fitted with a BDS Hypersil C18 column of dimensions 250 × 4.6 mm. Solvents utilised, in gradient mode, were analytical grade water acidified with 1% vol H_3_PO_4_ and HPLC grade acetonitrile.

The analytical protocol was operational under a constant flow rate (1.5 mL min^−1^) at a detector wavelength of 210 nm. Additionally, the column thermostat was fixed at 20 °C, ensuring a constant temperature throughout the analysis process. Calibration was carried out on a number of solutions of known concentration, with the standard error being found to be less that ±5%.

## 3. Results

### 3.1 The hydrogenation of mandelonitrile (MN)

**Scheme 2:**
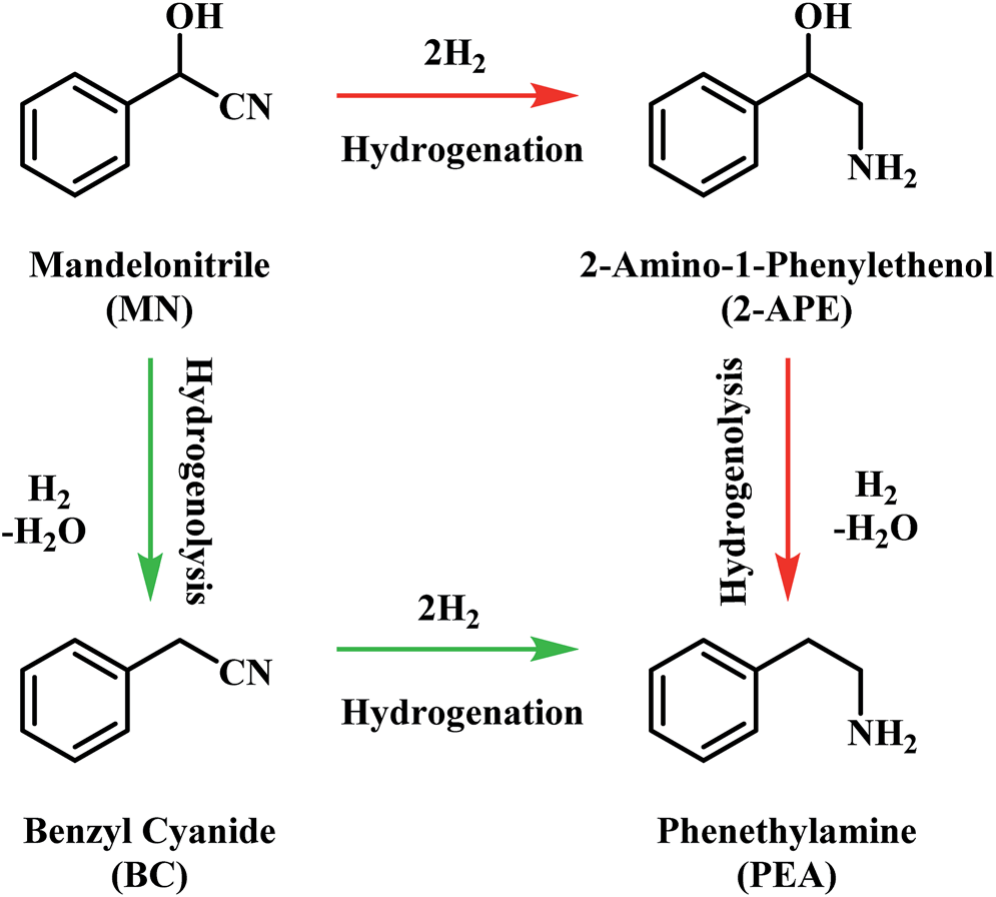
General reaction scheme for the hydrogenation of mandelonitrile showing the two available routes to phenethylamine.^25^

The pathway from cyanohydrin, mandelonitrile, to primary amine, phenethylamine, requires release of the hydroxyl group, a process that may be attributed to a dehydration reaction, forming water as a by-product. However, based on previous investigations by McMillan and co-workers into the hydrogenolysis of benzylamine, where the loss of ammonia was shown to be facilitated by a hydrogenolytic step, it is proposed that for the mandelonitrile system, it is a corresponding hydrogenolysis mechanism which facilitates the loss of water.^19,20^ Thus, with reference to Scheme 2, two possible routes to the desired product may be considered.^25^ Moreover, Scheme 2 highlights that the order in which the hydrogenation and hydrogenolysis steps take place dictates the intermediate species observed. With a thorough exploration of the hydrogenation of benzonitrile already undertaken,^19,20^ and as Scheme 2 suggests, a simple consecutive process from nitrile to target amine was initially envisaged.

### 3.2 Towards the production of phenethylamine

**Fig. 1:**
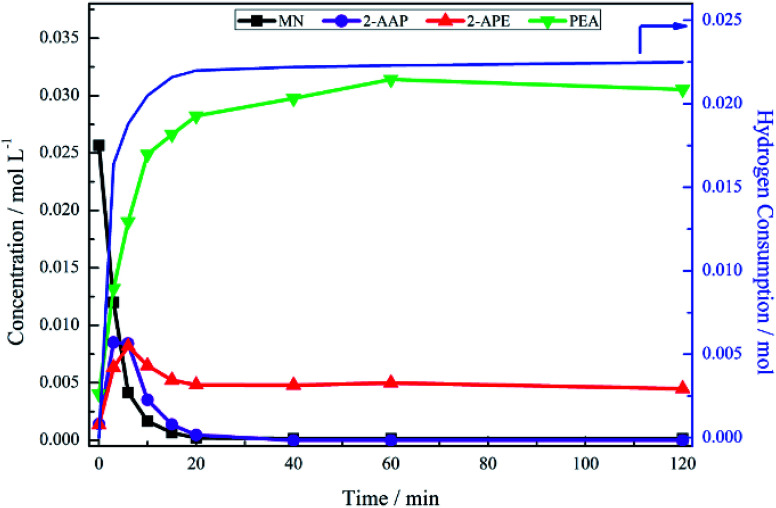
Reaction profile and hydrogen uptake curve for the hydrogenation of mandelonitrile over 300 mg 5% Pd/C catalyst, *ca.* 11 mmol mandelonitrile with 2 molar equivalents of H_2_SO_4_. Reaction carried out at 40 °C, 6 barg hydrogen with an agitation rate of 1050 rpm [MN = mandelonitrile; 2-AAP = 2-aminoacetophenone; 2-APE = 2-amino-1-phenylethanol; PEA = phenethylamine].

The hydrogenation of mandelonitrile was carried out under acidic conditions (Fig. 1). Similar to the previously studied 4-hydroxybenzyl cyanide hydrogenation system,^21^ the inclusion of an acid additive was found to be an essential component (Fig. S10†). In addition to the prevention of coupling reactions with the imine intermediate, the protonation of the amine functionality by the acid auxiliary is thought to prevent binding of the nitrogen lone pair to the surface of the catalyst and, thereby, acting as a poison. For the specific case of mandelonitrile, the acid presence allows an alternative binding motif, reversible in nature and *via* the hydroxyl group, ensuring that the reaction proceeds uninhibited. As can be observed in Fig. 1, full and rapid conversion of the nitrile moiety affords the desired product, phenethylamine. The accompanying hydrogen uptake curve shows cessation of hydrogen consumption at approximately 20 minutes, corresponding to complete conversion of the nitrile and a turnover number of 199 (6.62 × 10^21^ mandelonitrile molecules reacted over 3.33 × 10^19^ Pd surface atoms).

With reference to Scheme 2, the detection of 2-amino-1-phenylethanol and the absence of benzyl cyanide in the liquid phase indicates that hydrogenation precedes hydrogenolysis. Fig. 1, however, shows that residual quantities of the 2-amino-1-phenylethanol were present at reaction completion. Since no hydrogen consumption is observed beyond approximately 20 minutes, further reaction of the 2-amino-1-phenylethanol to yield phenethylamine is improbable after this point. This species is thus classed as a by-product of the reaction and cannot be considered as an intermediate, as was initially postulated in Scheme 2.

**Scheme 3:**
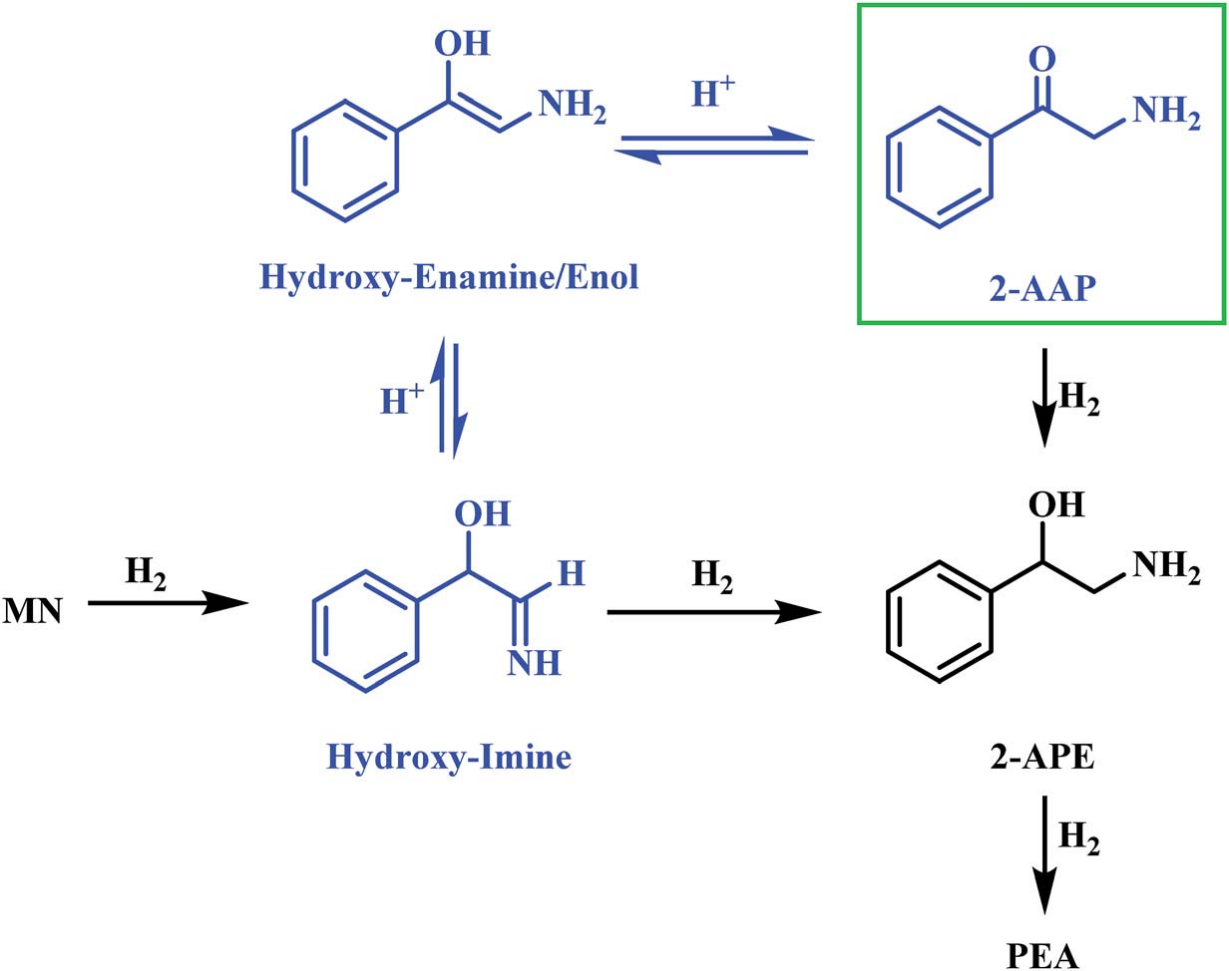
Proposed two step acid catalysed formation of 2-aminoacetophenone from the hydroxy-imine intermediate [MN = mandelonitrile; 2-AAP = 2-aminoacetophenone; 2-APE = 2-amino-1-phenylethanol; PEA = phenethylamine].

Alongside the production of phenethylamine and 2-amino-1-phenylethanol, another species was detectable in the liquid phase. Behaving as an intermediate (Fig. 1), this molecule was identified by LC-MS as the ketone 2-aminoacetophenone (2-AAP: C_6_H_5_C(O)CH_2_NH_2_). The formation of this species is acid catalysed, as evidenced by its absence under neutral conditions (Fig. S10†). The acid requirement provides rationalisation for the proposal of a tautomeric pathway in the mandelonitrile reaction system (Scheme 3). It is important to note at this juncture that no imine or enamine species are observable in the liquid phase, indicating that they exist as assumed adsorbed species. Within this constraint, their presence is implied and/or inferred within mechanistic considerations but, under the stated experimental arrangements, cannot be verified experimentally.

Referring to Scheme 3 it can be seen that the hydroxy-imine, which precedes the formation of 2-amino-1-phenylethanol, can undergo an acid catalysed tautomerisation to form 2-aminoacetophenone in a two-step process. The imine is first transformed to an enamine, which, due to the presence of the alcohol functionality can also be classified as an enol. This enol can then be converted to its keto form, 2-aminoacetophenone. This pathway is highlighted in blue in Scheme 3. Typically, at equilibrium, it is the keto form of the carbonyl species which is lower in energy,^24^ and thus predominates, demonstrating formation of 2-aminoacetophenone to be energetically favourable. Moreover, the acid catalysed nature of these tautomerisation reactions provides reasoning for the absence of 2-aminoacetophenone under neutral reaction conditions (Fig. S12†). Adding further complexity to the reaction system, it is acknowledged that, like 2-aminoacetophenone, the hydroxyl-imine and the hydroxyl-enamine are likely to be susceptible to hydrogenation and hydrogenolysis. Their existence as assumed adsorbed species further enhances this possibility.

**Fig. 2:**
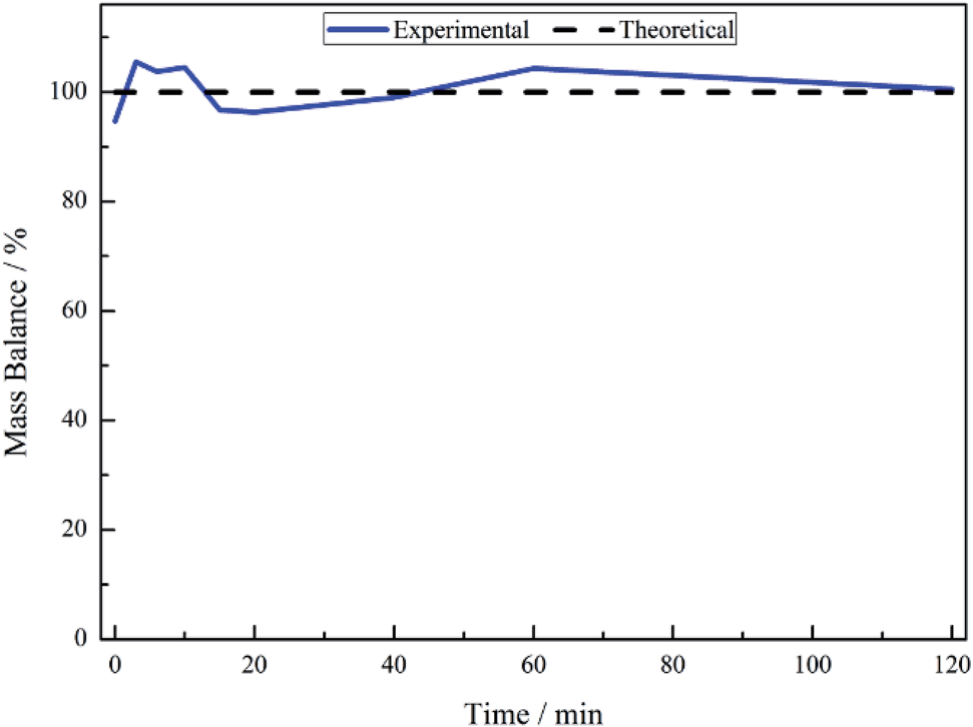
Mass balance plot for the hydrogenation of mandelonitrile over 300 mg 5% Pd/C catalyst, *ca.* 11 mmol mandelonitrile with 2 molar equivalents of H_2_SO_4_. Reaction carried out at 40 °C, 6 barg hydrogen with an agitation rate of 1050 rpm.

No other acid catalysed homogeneous reactions for the batch conditions presented here were detected, demonstrating a degree of control over the reaction as evidenced by a complete mass balance (Fig. 2). On completion of the reaction at approximately 40 minutes the selectivity towards, respectively, phenethylamine and 2-amino-1-phenylethanol was 87% and 13%.

### 3.3 Mechanistic considerations

Scheme 3 has highlighted the role of acid catalysed chemistry, which results in the formation of the intermediate 2-aminoacetophenone, in this reaction system. Consequently, the consecutive sequence of mandelonitrile → 2-amino-1-phenylethanol → phenylamine, as outlined in Scheme 2, is too simplistic and ultimately, unrepresentative of the associated liquid phase chemical transformations. In line with the complexity inferred from the chromatographic output, the potential pathways to afford phenethylamine must be considered in greater detail. As such, the hydrogenation/hydrogenolysis characteristics of 2-aminoacetophenone and 2-amino-1-phenylethanol were explored.

#### 3.3.1 The hydrogenolysis of 2-amino-1-phenylethanol (2-APE)

**Fig. 3:**
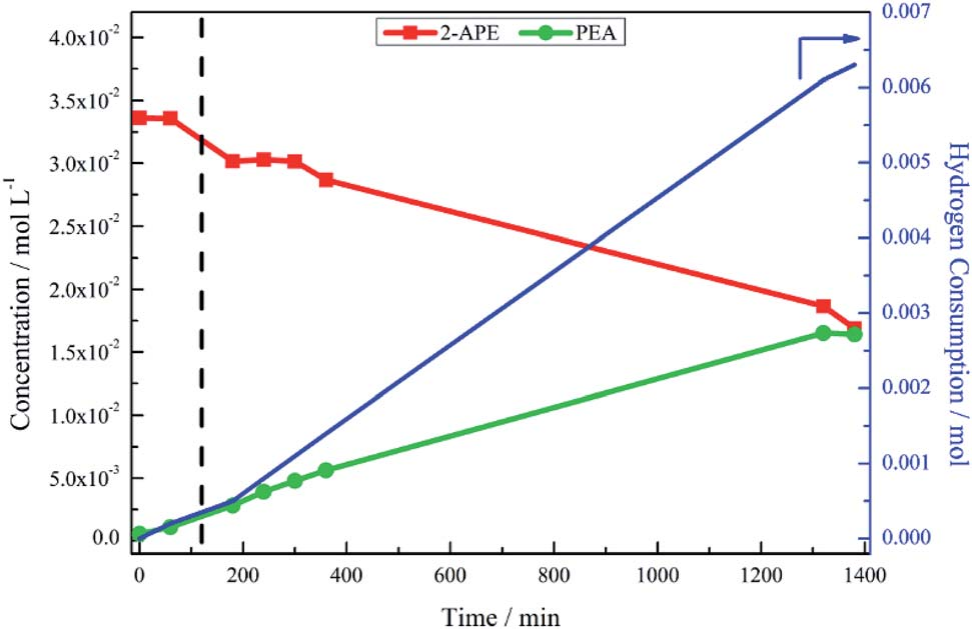
Reaction profile and hydrogen uptake curve for the hydrogenation of 2-amino-1-phenylethanol over 300 mg 5% Pd/C catalyst, *ca.* 11 mmol 2-amino-1-phenylethanol with 2 molar equivalents of H_2_SO_4_. Reaction carried out at 40 °C, 6 barg hydrogen with an agitation rate of 1050 rpm [2-APE = 2-amino-1-phenylethanol; PEA = phenethylamine]. 120 minutes along the *x*-axis is denoted by a dashed vertical line.

Fig. 3 presents the reaction profile for the hydrogenation of 2-amino-1-phenylethanol under standard reduction conditions over an extended period of time. As previously shown (Fig. 1), the conversion of the nitrile functionality was a relatively facile process with desired product formation, coinciding with cessation of hydrogen uptake, reaching a plateau at approximately 20 minutes into the reaction. It would thus be reasonable to assume that the hydrogenolysis of 2-amino-1-phenylethanol would occur on a similar time scale. Under the same set of reaction conditions, looking solely at the first 120 minutes of the reaction, however, Fig. 3 shows only minimal conversion of 2-amino-1-phenylethanol, resulting in a poor product yield (5%). Consistent with this constrained activity, Fig. 3 shows the hydrogen uptake at 120 min to be approximately 0.5 mmol H_2_, which corresponds to approximately 3% of the hydrogen uptake observed for full conversion of the same quantity of mandelonitrile reacted under the same conditions (Fig. 1).

It is possible that blockage of the active sites of the catalyst by the reagent is occurring, thereby indicating that 2-amino-1-phenylethanol acts as a catalyst poison. This possibility was evaluated by the addition of an aliquot of mandelonitrile and sulphuric acid to the reaction mixture at the two hour mark, without the introduction of any further catalyst. Analysis after the second addition of mandelonitrile (not presented) showed that the reaction proceeded as normal from this point. The rate of phenethylamine formation and yield was unaffected by the presence of the excess 2-amino-1-phenylethanol from the first part of the experiment. This observation eliminates the possibility of the hydroxyl amine poisoning catalyst active sites. It is therefore suggested that the route from 2-amino-1-phenylethanol to phenethylamine is kinetically slow when compared to other stages of the overall process.

The hypothesis of the transformation of 2-amino-1-phenylethanol to phenethylamine occurring at a leisurely pace was confirmed, again with reference to Fig. 3, where the reaction was monitored for a further 21 hours. As the reaction progressed, there was increased hydrogen consumption as well as the slow conversion of the 2-amino-1-phenylethanol to phenethylamine. At 23 hours 2-amino-1-phenylethanol conversion was 46%, with an accompanying phenethylamine yield of 48%. Table 1 compares the yield of phenethylamine at *t* = 20 minutes for the hydrogenation of 2-amino-1-phenylethanol (0.9%) with the hydrogenation of mandelonitrile (81%). The significantly reduced phenethylamine yield associated with the hydrogenolysis of 2-amino-1-phenylethanol indicates this transformation to be unfavourable. Moreover, within the 23 hour monitoring time (Fig. 3), complete conversion of the 2-amino-1-phenylethanol was not accomplished.

**Table 1:** Conversion of starting material (mandelonitrile; 2-amino-1-phenylethanol; 2-aminoacetophenone) and yield of phenethylamine at *t* = 20 minutes. Reactions carried out at 40 °C, 6 barg hydrogen with an agitation rate of 1050 rpm. In all instances 300 mg 5% Pd/C catalyst was used for the hydrogenation of 11 mmol mandelonitrile and 2-amino-1-phenylethanol but 2.75 mmol 2-aminoacetophenone. Two molar equivalents of H_2_SO_4_ with respect to the concentration of starting material was also included in the reaction mixture

Starting material	Conversion at *t* = 20 min	Phenethylamine yield at *t* = 20 min
Mandelonitrile	100%	81%
2-Amino-1-phenylethanol	1.1%	0.9%
2-Aminoacetophenone	100%	12%

The observed build-up of 2-amino-1-phenylethanol in the reaction mixture after two hours (Fig. 1) can therefore be rationalised by the slow kinetics of this hydrogenolysis process. Further, whilst Fig. 3 shows that hydrogenolysis of 2-amino-1-phenylethanol does indeed produce phenethylamine, it also shows that the reaction is too slow to make a significant contribution to product formation within the timescale of the mandelonitrile hydrogenation reaction (approximately 40 minutes). Therefore, there must be an alternative chemical pathway, or pathways, that are responsible for accessing phenethylamine.

#### 3.3.2 Hydrogenation of 2-aminoacetophenone (2-AAP)

The identification of 2-aminoacetophenenone as an intermediate species in the hydrogenation of mandelonitrile is evidenced by the reaction profile presented in Fig. 1. The role of 2-aminoacetophenone in the overall reaction process must now be considered. Scheme 3 indicates that, in a hydrogenating environment, the ketone functionality of 2-aminoacetophenone will be hydrogenated to the corresponding alcohol forming 2-amino-1-phenylethanol.^43–45^ From 2-amino-1-phenylethanol, it would then be expected that hydrogenolysis to phenethylamine would occur. However, the preceding section (Section 3.2.1) indicates the hydrogenolysis of 2-amino-1-phenylethanol to form the desired product to be kinetically unviable.

**Scheme 4:**
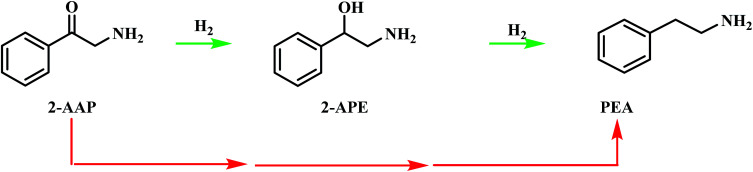
Postulated pathways for the conversion of 2-aminoacetophenone (2-AAP) to the primary amine phenethylamine (PEA). The red arrows signify an unspecified, multi-step pathway.

Consequently, another route from 2-aminoacetophenone to phenethylamine, that does not proceed *via* 2-amino-1-phenylethanol, must be considered. This alternative route is signified by the red arrows in Scheme 4. It should be noted that this pathway may proceed *via* a number of species and does not indicate direct hydrogenolysis of the carbonyl group. In order to achieve greater mechanistic clarity, the hydrogenation of 2-aminoacetophenone was undertaken to aid the elucidation of the role of this species in the reaction's progression to phenethylamine.

The presence of an acid additive has been shown to be an essential component of the reaction mixture (Section 3.2 and Fig. S10†) and therefore it is prudent to establish the role of the acid. 2-Aminoacetophenone is commercially available as the 2-aminoacetophenone hydrochloride salt (Section 2.1), providing the opportunity to explore its catalytic conversion both in the absence and in the presence of the sulphuric acid auxiliary agent. As formerly described in Section 3.1, the acid additive was employed in the hydrogenation of mandelonitrile to protonate the amine functionality, thus preventing the strong binding of the nitrogen lone pair to the surface of the catalyst, which can result in catalyst deactivation.^37,46^ Accordingly, the requirement for sulphuric acid for protonation purposes becomes obsolete when the species is present as a salt. This additionally allows the effect of the acid to be mimicked without the contribution of its inherent catalytic activity.

**Scheme 5:**
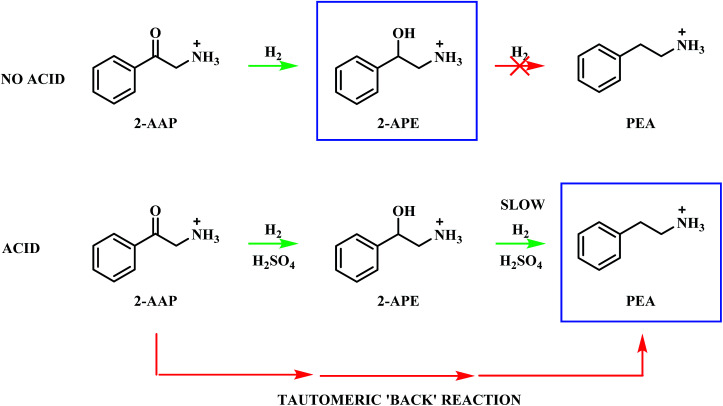
Schematic representation of the species detectable in the liquid phase for the hydrogenation of 2.75 mmol 2-aminoacetophenone hydrochloride over 300 mg 5% Pd/C catalyst, in MeOH both with and without two molar equivalents of H_2_SO_4_. Reaction carried out at 40 °C, 6 barg hydrogen with an agitation rate of 1050 rpm. The final product of each reaction is highlighted by a blue box.

Upon exploration of the aforementioned experimental conditions, vastly different profiles were obtained (Fig. S12 and S13†). The results of the experimental findings for the hydrogenation of 2-aminoacetophenone are summarised in Scheme 5. Under both described conditions the 2-aminoacetophenone salt was rapidly hydrogenated to 2-amino-1-phenylethanol (*t* ≈ 3 minutes), indicating this process to be highly favourable and independent of the catalytic powers of the acid. The accelerated nature of this transformation meant that no accurate rate data could be obtained. For the neutral environment (Fig. S12†) the reaction reached completion with 2-amino-1-phenylethanol as the sole detectable product, thus ruling out the possibility of direct hydrogenolysis of the ketone to the target amine in the forward direction (Scheme 4).^44,45,47^

The addition of acid to the reaction mixture, however, afforded a very different profile (Fig. S13†). Phenethylamine production is shown to be facilitated by the acid additive, with this species detectable alongside 2-amino-1-phenylethanol at the end of the reaction. Continuous hydrogen consumption was evident throughout the reaction. Interestingly, the yield of phenethylamine at *t* = 20 minutes for this reaction (12%, Table 1) was higher than the yield obtained for the direct hydrogenation of 2-amino-1-phenylethanol (0.9%, Table 1). With reference to Scheme 5, these results establish that, in addition to preventing amine groups from site blocking at the Pd surface, the sulphuric acid additionally facilitates phenethylamine production. It is proposed that the reversible nature of the tautomeric equilibria between the hydroxy-imine and the hydroxy-enamine, and the hydroxy-enamine and the 2-aminoacetophenone,^24^ will provide a tautomeric route, in the reverse direction, from the 2-aminoacetophenone to phenethylamine.

## 4. Discussion

The reaction profile for mandelonitrile hydrogenation over a Pd/C catalyst identifies phenethylamine as the principle product (selectivity 87%), 2-amino-1-phenylethanol as a by-product (selectivity 13%) and 2-aminoacetophenone as a reaction intermediate (Fig. 1). The reaction sequence does not conform to a simple consecutive reaction as envisaged in Scheme 2, not least because Section 3.3.1 establishes that the hydrogenolysis of 2-amino-1-phenylethanol to form phenethylamine is kinetically slow. Moreover, Section 3.3.2 shows that under the specified reaction conditions, explicitly in the presence of sulphuric acid as an auxiliary agent, the desired primary amine product (phenethylamine) can be produced from 2-aminoacetophenone under conditions of continuous hydrogen consumption. The above considerations involve all the chemical entities detectable by HPLC. Further mechanism development, however, is thwarted by the participation of reactive species, assumed to be adsorbed, and thus not observable in the liquid phase. One now needs to consider additional chemical pathways that can account for the reaction profile presented in Fig. 1.

Section 3.2 introduced the availability of a tautomeric pathway, originating at the hydroxy-imine entity, and culminating at 2-aminoacetophenone (Scheme 3). Whilst not detectable chromatographically, the hydroxy-imine and hydroxy-enamine species are believed to be essential intermediates in the production of 2-aminoacetophenone. As mentioned prior, as with 2-aminoacetophenone, both the hydroxy-imine and hydroxy-enamine are chemically susceptible to both hydrogenation and hydrogenolysis. Therefore, in order to construct a reaction scheme that contains chemically viable reaction pathways, one needs to take account of the following six deductions/observations: (i) mandelonitrile hydrogenation is fast (Section 3.2); (ii) the hydrogenation of 2-aminoacetophenone to produce 2-amino-1-phenylethanol is fast (Section 3.3.2); (iii) the hydrogenolysis of 2-amino-1-phenylethanol to produce phenethylamine is prohibitively slow (Section 3.3.1); (iv) 2-amino-1-phenylethanol is a by-product (Section 3.2); (v) 2-aminoacetophenone is a reaction intermediate, the formation of which is associated with a reversible, acid catalysed tautomeric pathway (Section 3.2); (vi) the hydroxy-imine and hydroxy-enamine (enol) entities could undergo direct hydrogenation to form 2-amino-1-phenylethanol (Section 3.2).

**Scheme 6:**
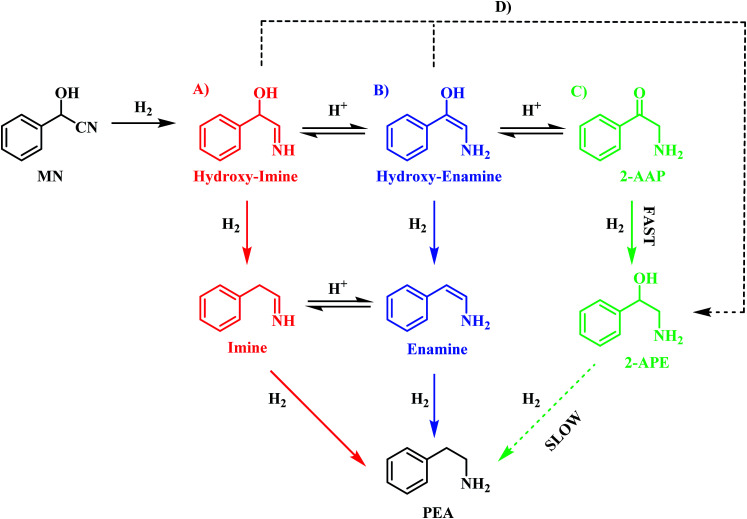
Proposed reaction scheme for the hydrogenation of mandelonitrile over Pd/C. Scheme includes the intermediate 2-aminoacetophenone and highlights that there are multiple postulated pathways (A–D) to phenethylamine [MN = mandelonitrile; 2-AAP = 2-aminoacetophenone; 2-APE = 2-amino-1-phenylethanol; PEA = phenethylamine]. It is noted that the hydroxy-imine/hydroxy-enamine and imine/enamine entities are present as postulated adsorbed species, whilst MN, 2-AAP, 2-APE and PEA are partitioned between solvated and adsorbed species at the liquid/solid interface.

On the basis of this perspective, Scheme 6 is tentatively proposed as defining a global reaction scheme for the hydrogenation of mandelonitrile over a Pd/C catalyst to produce the primary amine phenethylamine. Scheme 6 indicates that, far from the simple consecutive pathway initially envisaged (Scheme 2), there are now at least four potential routes from reagent to desired product. The four main pathways, highlighted as A, B, C and D in Scheme 6, include three direct forward pathways originating from each of the tautomeric equilibria species designated in Scheme 3 (hydroxy-imine, A; hydroxy-enamine, B and 2-aminoacetophenone, C). It is proposed that the hydroxy-imine and hydroxy-enamine undergo hydrogenolysis to form the corresponding imine and enamine species, before undergoing a final hydrogenation step to afford phenethylamine in both cases. Pathway D represents the potential for direct hydrogenation of either the hydroxy-imine or hydroxy-enamine to 2-amino-1-phenylethanol, where the concluding step would be hydrogenolysis of the hydroxyl functionality to yield phenethylamine, as with path C.

Prior investigation has indicated that routes C and D are unlikely to be significant contributors to phenethylamine production due to the 2-amino-1-phenylethanol hydrogenolysis bottle-neck (Section 3.3.1). Indeed, it is this sluggish transformation which results in formation of 2-amino-1-phenylethanol as a by-product of the reaction. Removal of paths C and D leaves routes A and B as potential candidates for the most likely routes by which mandelonitrile can be converted to phenethylamine. In addition to the reaction proceeding exclusively in the forward direction, as is depicted in Scheme 6, the reversible nature of the tautomeric equilibria provides an additional opportunity for routes A and B to be followed. Specifically, it is proposed that, upon formation of the energetically favourable 2-aminoacetophenone, the tautomeric equilibrium shifts back to reform the hydroxy-enamine and hydroxy-imine species. From here, routes A and B can be followed to furnish phenethylamine as previously described. Against this reasoning, it is proposed that the reaction proceeds *via* either pathway A and/or pathway B.

Unfortunately, pathways A and B cannot be differentiated using the chromatographic methods presented here, as the corresponding intermediates are undetectable in the liquid phase. It is therefore proposed that in the hydrogenation of mandelonitrile, phenethylamine formation has the potential to proceed *via* either one or both of these routes. Further investigation into the more conclusive identification of the active pathway or pathways involved in this reaction constitutes ‘work in progress’.

## 5. Conclusions

The liquid phase hydrogenation of mandelonitrile over a 5% Pd/C catalyst has been investigated. An acid additive proved to be not only essential for sustained catalytic activity (Section 3.1), but also played a vital role in catalysing the homogeneous reaction responsible for formation of the ketone intermediate 2-aminoacetophenone (Section 3.2). The resultant tautomeric pathway is thought to open-up additional routes that could subsequently aid phenethylamine formation. A tentative global reaction scheme is proposed (Scheme 6) to account for the pathways accessible within this chemical network; the scheme includes contributions from species assumed to be solely adsorbed as well as entities that are partitioned between solvated and adsorbed states at the liquid/solid interface.

For a single batch run, successful production of the primary amine, phenethylamine, was achieved with full conversion (*X*_mandelonitrile_ = 100%; *S*_phenethylamine_ = 87%, Fig. 1). Production of 2-amino-1-phenylethanol accounts for the remaining 13% selectivity and completes the mass balance (Fig. 2). When the kinetics of the conversion of the proposed intermediate 2-amino-1-phenylethanol were considered, it was found that this process was significantly slower than the overall production of phenethylamine. This finding excluded the possibility that 2-amino-1-phenylethanol is an intermediate and is instead designated as a by-product of the reaction. Moreover, it was indicated that an alternative route from reagent to product must be operational. Consequently it is proposed that the hydrogenolytic cleavage of the C–OH bond occurs on either one, or both, of the highly reactive hydroxy-imine and hydroxy-enamine species. Differentiation between these two proposed pathways, however, was not possible by the chromatographic procedures employed here.

## Conflicts of interest

There are no conflicts to declare.

## Supplementary Material

RA-009-C9RA04618F-s001
