# Supplementary material for: The hydrogenation of mandelonitrile over a Pd/C catalyst: towards a mechanistic understanding
Source: RSC Adv. 2019 Aug 21;9(45):26116–25. doi: 10.1039/c9ra04618f (PMC9070305; doi:10.1039/c9ra04618f)
Supplement: RA-009-C9RA04618F-s001 [file RA-009-C9RA04618F-s001.pdf]

Electronic Supplementary Material (ESI) for RSC Advances.
This journal is © The Royal Society of Chemistry 2019

Electronic Supplementary Information

The Hydrogenation of Mandelonitrile over a Pd/C Catalyst: Towards a Mechanistic Understanding

Mairi I. McAllister[a], Cédric Boulho[a], Liam McMillan[a], Lauren F. Gilpin[a], Colin Brennan[b] and David Lennon*[a]

[a] School of Chemistry, Joseph Black Building, University of Glasgow, Glasgow, G12 8QQ, UK.

[b] Syngenta, Jeallot's Hill International Research Centre, Bracknell, Berkshire, RG42 6EY, UK.

Further Catalyst Characterisation Details

Nitrogen Physisorption

Transmission Electron Microscopy

Scanning Electron Microscopy and Energy Dispersive X-ray Spectroscopy

Powder X-Ray Diffraction

Raman Spectroscopy

CO Chemisorption

Thermogravimetric Analysis

The Hydrogenation of Mandelonitrile Under Neutral Conditions

The Quenching of Unwanted Homogeneous Chemistry Leading to Control of the Reaction Profile

The Hydrogenation of 2-Aminoacetophenone

References

Further Catalyst Characterisation Details

Nitrogen Physisorption

Specific surface areas and textural data were measured on a Quantachrome Evo instrument after degassing (overnight *ca.* 20 hours) on a Quantachrome FloVa degasser. Data anaylsis was conducted using the Quadrawin software package.

Nitrogen physisorption of the catalyst revealed a specific surface area of 775 ±19 m^2g^{-1}. The isotherm, Figure S1, can be defined as type IV with an accompanying hysteresis loop associated with the occurrence of pore condensation.[a] The hysteresis loop is identified as H4 whcih is indicative of narrow, slit pores or pores within the microporous region.[b]

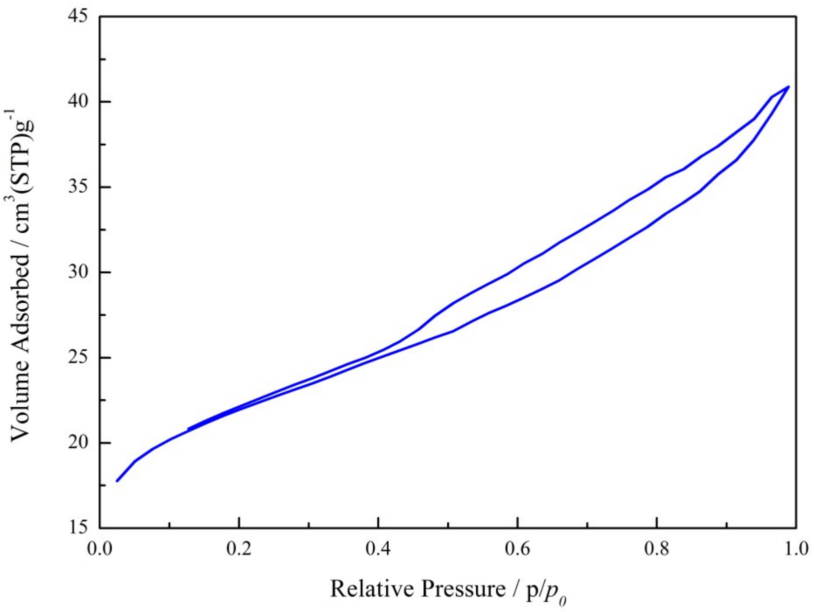

Figure S1. *Nitrogen adsorption isotherm for the 5% Pd/C catalyst used for the hydrogenation of mandelonitrile.*

Transmission Electron Microscopy

TEM analysis was conducted using a Tecnai G2T20 STWIN fitted with a tungsten filament operating at an acceleration voltage of 200 KV. Representative TEM micrographs can be seen in Figure S2.

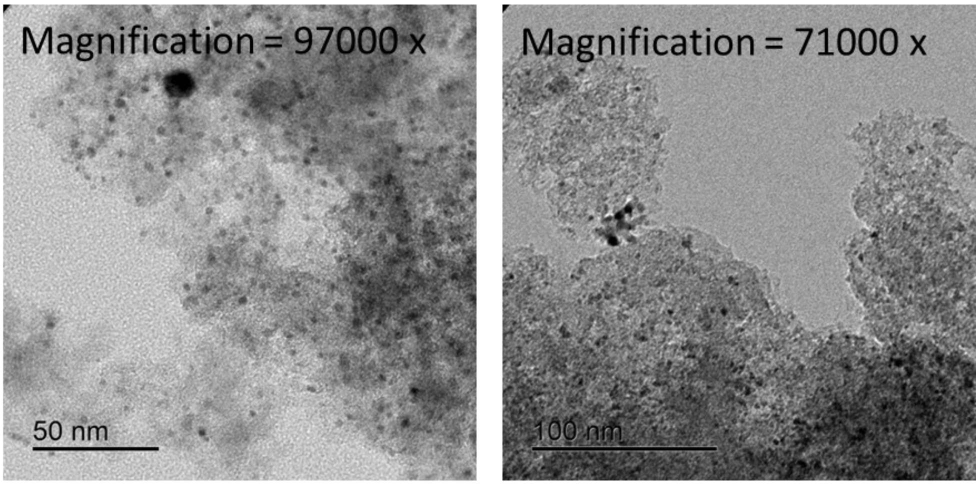

Figure S2. *Representative TEM micrographs for the 5% Pd/C catalyst used for the hydrogenation of mandelonitrile.*

A particle size distribution histogram (Figure S3) reveals that the diameter of the majority of the Pd particles is in the range of 2–3 nm.

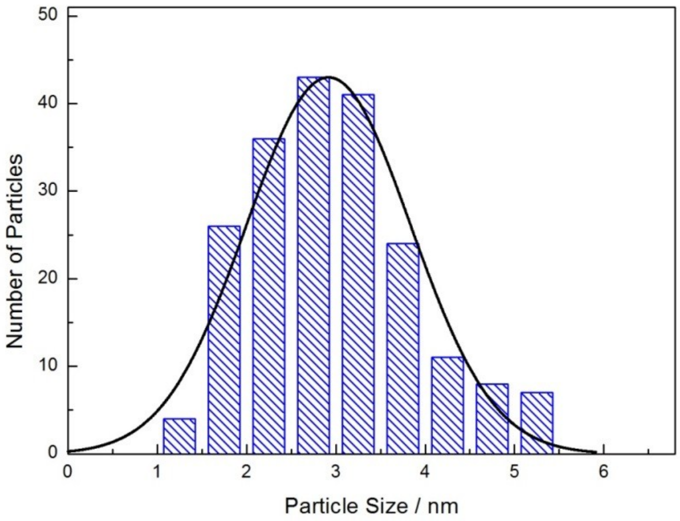

Figure S3. *Particle size distribution histogram for the 5% Pd/C catalyst as determined by a particles size count (total of 200 particles) of the corresponding TEM images using the Gatan Microscopy Suite Software. The solid black line represents a Gaussian fitting.*

Scanning Electron Microscopy and Energy Dispersive X-ray Spectroscopy

Scanning electron microscopy was carried out on the catalyst using a Philips XL30 ESEM instrument with an Oxford instruments X-act spectrometer for EDX measurements. The EDX was calibrated using the INCA EDX software with Cu as the calibrated standard. The sample was coated in gold using a sputter coating procedure to improve the contrast. Representative SEM micrographs are presented in Figure S4. The micrographs show the carbon supported catalyst to be composed of irregular plate-like structures ranging in size from approximately 4 µm to approximately 55 µm.

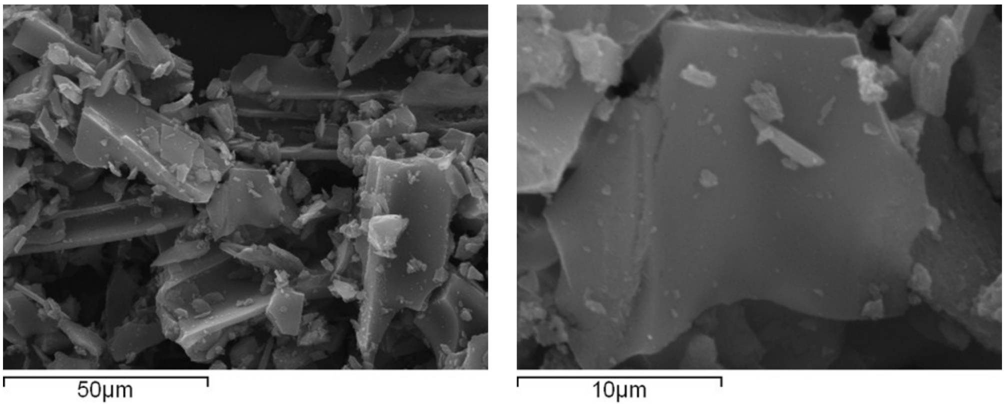

Figure S4. *Representative SEM micrographs for the 5% Pd/C catalyst used for the hydrogenation of mandelonitrile.*

The corresponding energy dispersive x-ray spectroscopy allowed the distribution of the elements within the sample to be mapped (Figure S5). From this method, the percentage loading of the Pd on the catalyst was found, from two areas, to be 3.12 ± 1.2%.

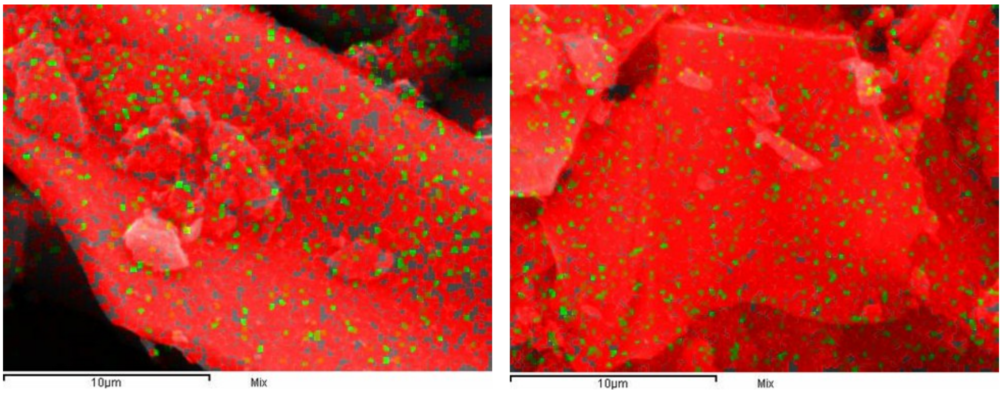

Figure S5. *Representative EDX maps for the 5% Pd/C catalyst used for the hydrogenation of mandelonitrile. [C red; Pd green].*

Powder X-Ray Diffraction

Powder x-ray diffraction was carried out on the 5% Pd/C catalyst using a Panalytical X'Pert PRO MPD diffractometer equipped with a Cu sealed X-ray source in the 2θ 5–85° with a step size of 0.017°. The resultant PXRD pattern can be visualised in Figure S6 alongside reference patterns as calculated by the ICSD for both carbon (card number: 76767) and palladium (card number: 52251). Figure S6 largely shows the amorphous nature of the carbon support, with negligible contribution from the Pd crystallites.

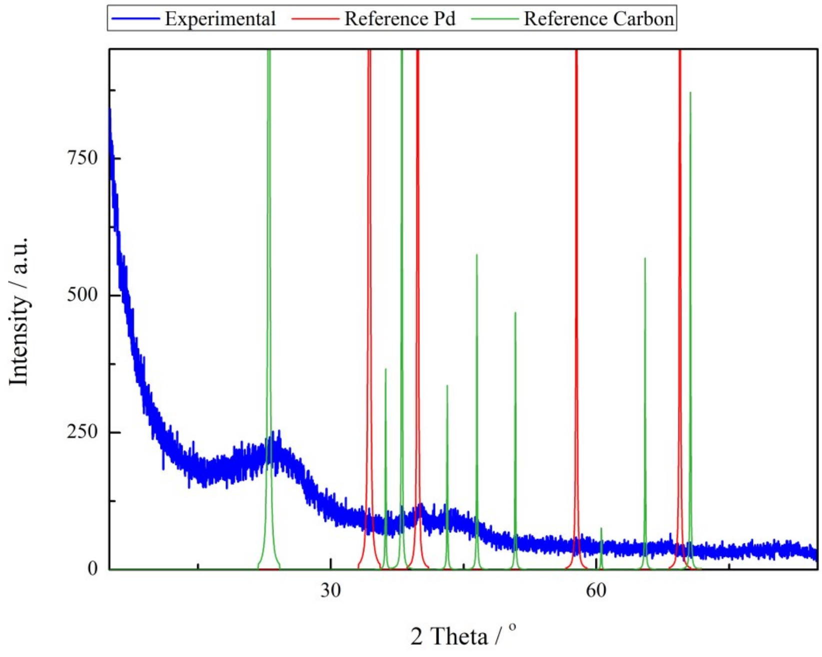

Figure S6. *Powder X-ray diffraction pattern, including reference Pd and carbon patterns for the 5% Pd/C catalyst used for the hydrogenation of mandelonitrile.*

Raman Spectroscopy

Raman spectroscopy was employed using a Horiba Jobin-Yvon LabRam Raman HR800 operating with a 514 nm laser. An aperture size of 100 µm and a 1% filter was used in order to prevent sample degradation.

The resultant Raman spectrum is indicative of carbonaceous material: disordered carbon (D1, D2, D4), amorphous carbon (D3) and ordered carbon (G). Curve fitting of the Raman spectrum (Figure S7) affords the following assignments: D1 (1348 cm^{-1}), D3 (1508 cm^{-1}), D4 (1182 cm^{-1}) and G (1061 cm^{-1}).

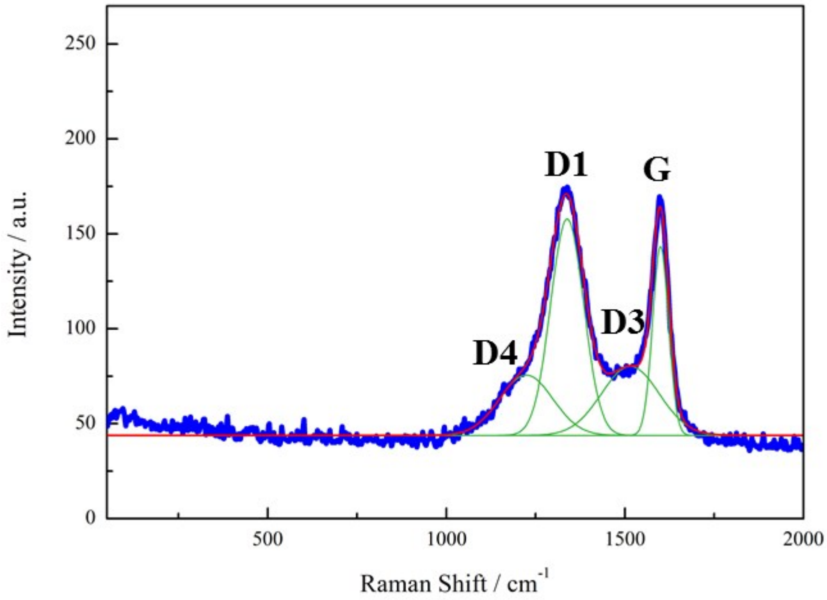

Figure S7. *Ramam spectrum for the 5% Pd/C catalyst used for the hydrogenation of mandelonitrile. The spectrum is fitted by a combination of four Gaussian line shapes (green lines), with the red line representing the cumulative fit and the blue line indicating the experimental data. It should be noted that D2 has not been fitted due to observational difficulties.*[c]

CO Chemisorption

Pulsed carbon monoxide chemisorption measurements were carried out in a quarz 'u-tube' reactor. A known volume of CO was pulsed over the catalyst with saturation points measured by gas chromatography (Thermo Finnigan Ultra GC fitted with a thermal conductivity detector). A maximum CO capacity of 9.24 $\times 10^{-5}$ mol CO/$g_{catalyst}$ was obtained. If one assumes a CO:Pd(s) ratio of 1:2 this equates to 1.11 $\times 10^{20}$ Pd(s) atoms/$g_{catalyst}$, which corresponds to a Pd dispersion of 39% and a mean particle size of 2.77 nm. A representative isotherm is shown in Figure S8.

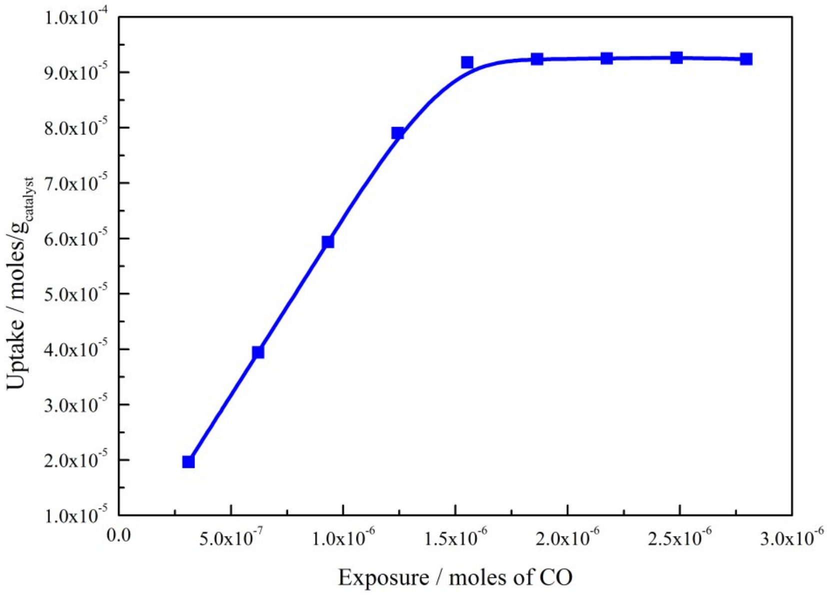

Figure S8. *Representative CO chemisorption isotherm for the 5% Pd/C catalyst used for the hydrogenation of mandelonitrile.*

Thermogravimetric Analysis

The thermal stability of the catalyst was determined by thermogravimetric analysis (Figure S9). The temperature was ramped to 1000 °C at 10 °C min^{-1} in argon. The measurement was carried out using a SDT Q600 V8.3 Build 101. Figure S9 shows that the catalyst is not stable at high temperatures as evidenced by a degree of decomposition. As such, the reaction must operate under stable temperatures to ensure maximum efficiency.

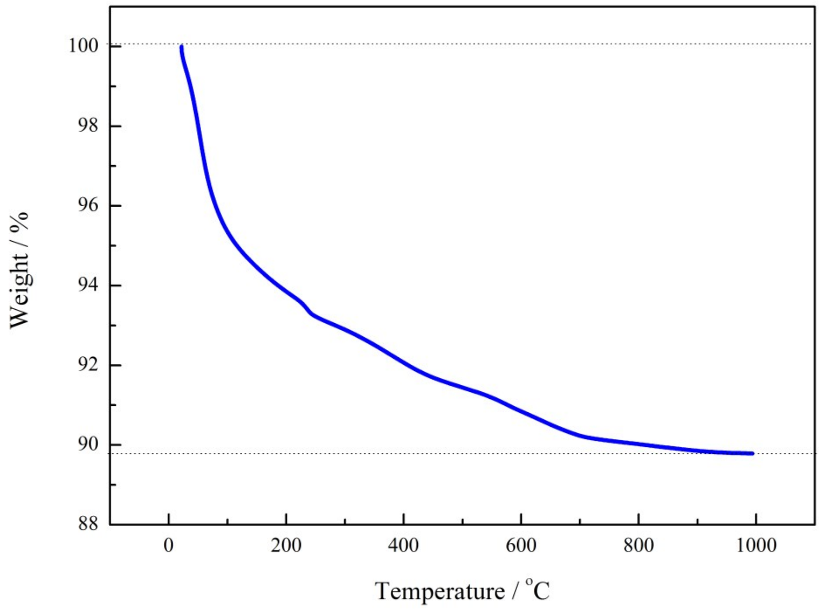

Figure S9. *Thermogravimetric analysis of the 5% Pd/C catalyst used for the hydrogenation of mandelonitrile.*

The Hydrogenation of Mandelonitrile Under Neutral Conditions

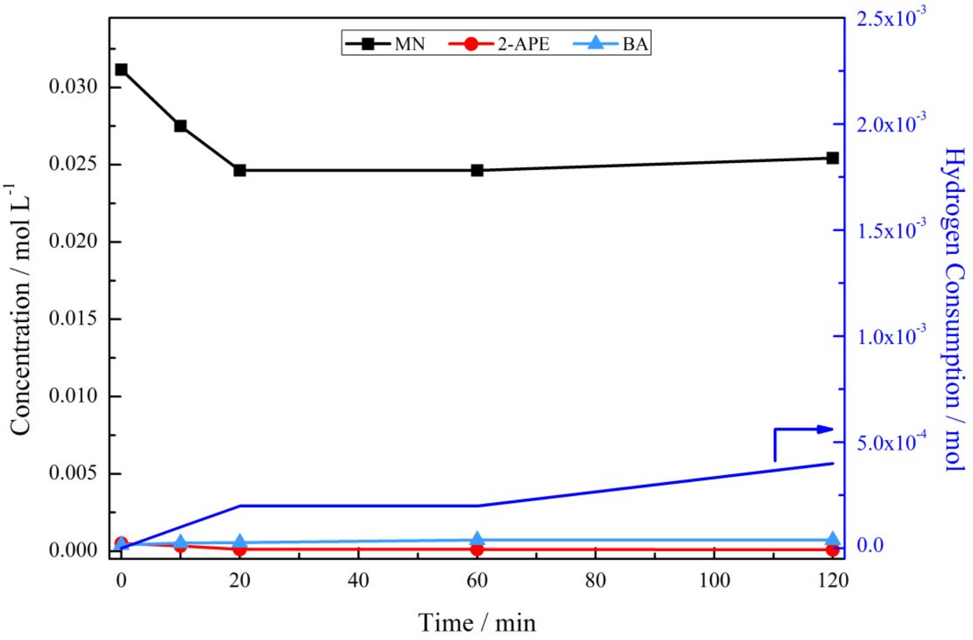

Figure S10. *Reaction profile and hydrogen consumption curve for the hydrogenation of mandelonitrile over 300 mg 5% Pd/C catalyst with ca. 11 mmoles mandelonitrile. Reaction carried out at 40 °C, 6 barg hydrogen with an agitation rate of 1050 r.p.m.. [MN = mandelonitrile; 2-APE = 2-amino-1-phenylethanol; BA = benzylalcohol].*

Initial studies into the hydrogenation of mandelonitrile were carried out under neutral conditions (Figure S10). The results, however, were not favourable, with no detection of the primary amine product. Figure S10 also shows production of trace quantities of the hydroxyl-amine, 2-amino-1-phenylethanol, and a significantly reduced hydrogen uptake than expected for full conversion of the nitrile.

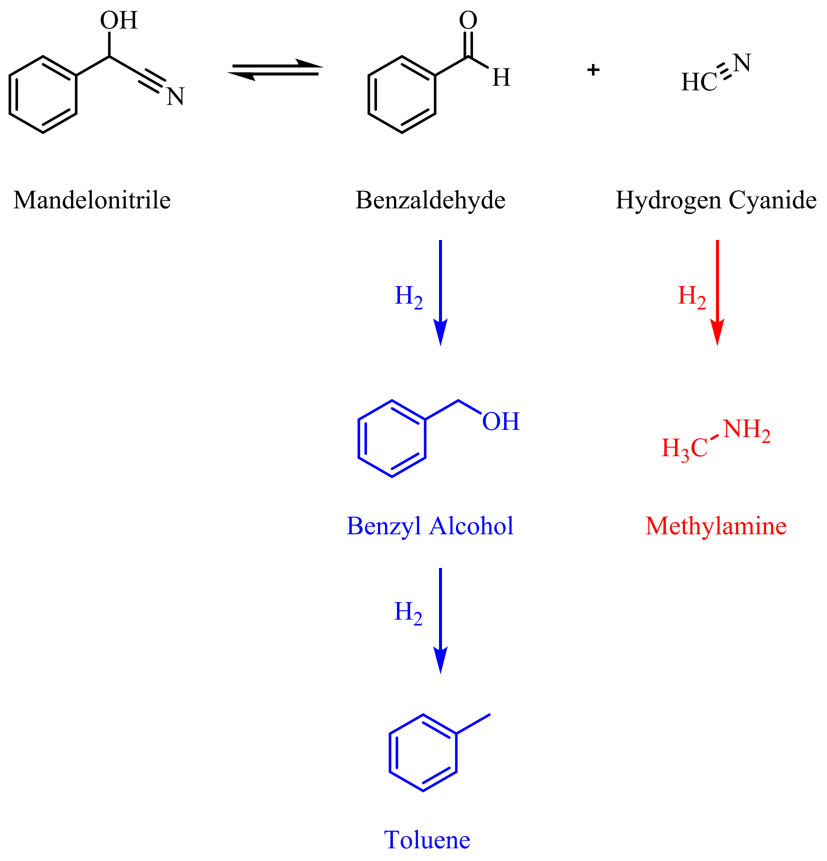

Scheme S1. *Proposed reaction scheme for the hydrogenation of mandelonitrile under neutral conditions.*

Furthermore, and rather unprecedented, was consumption of the starting material far in excess of detectable product yield. As a direct result of this observation, a mass imbalance was detected as the reaction progressed. Literature reports that under neutral conditions mandelonitrile is not stable and is susceptible to spontaneous decomposition to its starting materials: benzaldehyde and hydrogen cyanide.[d] Whilst benzaldehyde is measurable by HPLC, it was found to have a very similar elution time to mandelonitrile, making it challenging to detect if this process was occurring in the reaction system. However, as there was hydrogen available in the reaction vessel it is possible for the benzaldehyde to be hydrogenated, first to benzyl alcohol (BA) and then to toluene. The suggested reaction routes for these reactions can be seen in Scheme S1. Supporting this hypothesis is the production, albeit in negligible quantities, of benzyl alcohol (Figure S10). Unfortunately, toluene was unidentifiable using the

HPLC method employed. Nevertheless, the undetectable nature of this species can be used to provide a potential reason for the loss in mass observed at the end of the reaction. Expected alongside this reaction would therefore be the production of the highly undesirable hydrogen cyanide. It is possible that this species could then be hydrogenated to methylamine which, as a gaseous species, would not be detectable in the liquid phase.[e] Precautions taken to prevent the release of the harmful hydrogen cyanide are detailed in the experimental section (Section 2.2).

The Quenching of Unwanted Homogeneous Chemistry Leading to Control of the Reaction Profile

The occurrence of undesirable homogeneous reactions was found to complicate the analysis method giving misleading results. Analysis of the reaction mixture at ambient temperature, however, revealed a peak area inconsistency at the elution time of 2-amino-1-phenylethanol that was shown to be time dependent. Subsequent investigation of sample stability was undertaking by taking a large aliquot of reaction mixture from a standard mandelonitrile hydrogenation reaction at a time where the species, identified as the source of the variable peak output, was known to be present. Residual catalyst was then filtered off and the sample divided into equal parts. Each vial was then placed in the HPLC auto-sampler with a time delay between the analyses of each sample. This allowed the area of the 2-amino-1-phenylethanol peak to be measured with respect to time. As each vial contained a sample taken from the autoclave at the same time, each chromatograph should have produced an identical trace. Peak area, however, was shown not to be constant (Figure S11) and, instead, increased with time indicating that the reaction mixture was not stable at room temperature.

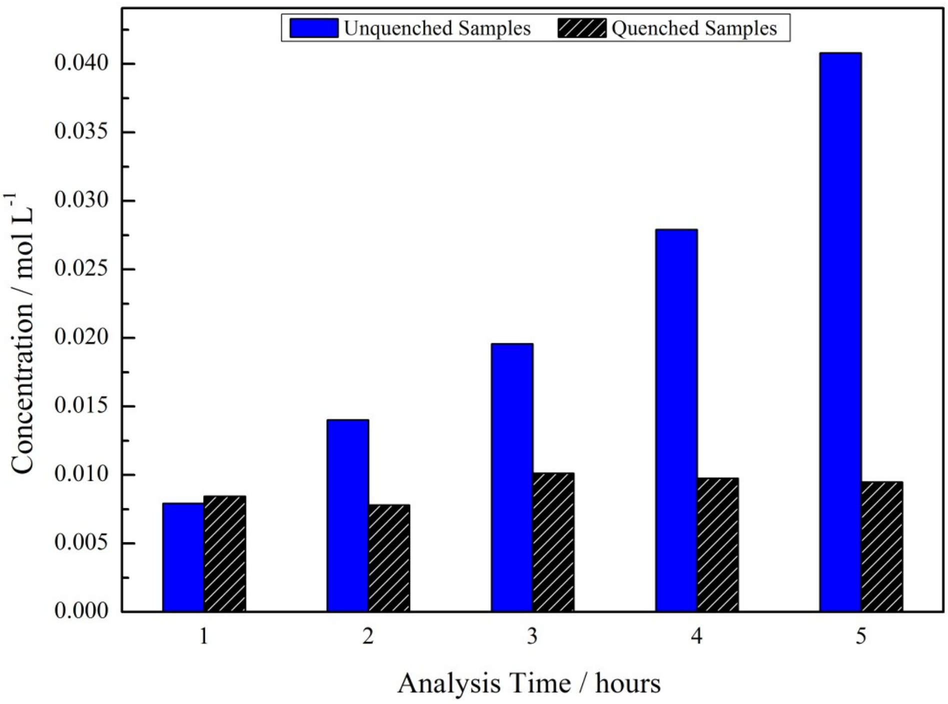

Figure S11. *Concentration values obtained chromatographically for the '2-amino-1-phenylethanol' peak at various times post-extraction from the autoclave comparing the quenched and unquenched samples. Extraction time increases linearly with reaction sample.*

It was proposed that it was the occurrence of homogeneous chemistry in the sample vials at ambient temperatures, in the form of acid catalysed reactions, which resulted in the peak growth observed. Specific reactions taking place under these conditions were not identified but their occurrence indicates the existence of underlying complexity in the homogeneous phase of this reaction system. Ensuing alteration of the experimental protocol to involve the quenching of all reaction sample vials in ice prior to HPLC analysis alleviated this problem with a much more consistent concentration being obtained (Figure S11 quenched sample bars).

For a single batch run the issues associated with homogeneous chemistry were eliminated, utilising HPLC analysis of quenched samples, with the resultant profile being representative of the hydrogenation reaction.

The Hydrogenation of 2-Aminoacetophenone

The profile for the hydrogenation of 2-aminoacetophenone is affected by the absence or presence of an acid additive. Due to the rapid nature of the conversion, before sampling, the 2-aminoacetophenone is nearly completely reacted in the neutral environment (Figure S12); for acidic conditions, all of the reagent is consumed (Figure S13).

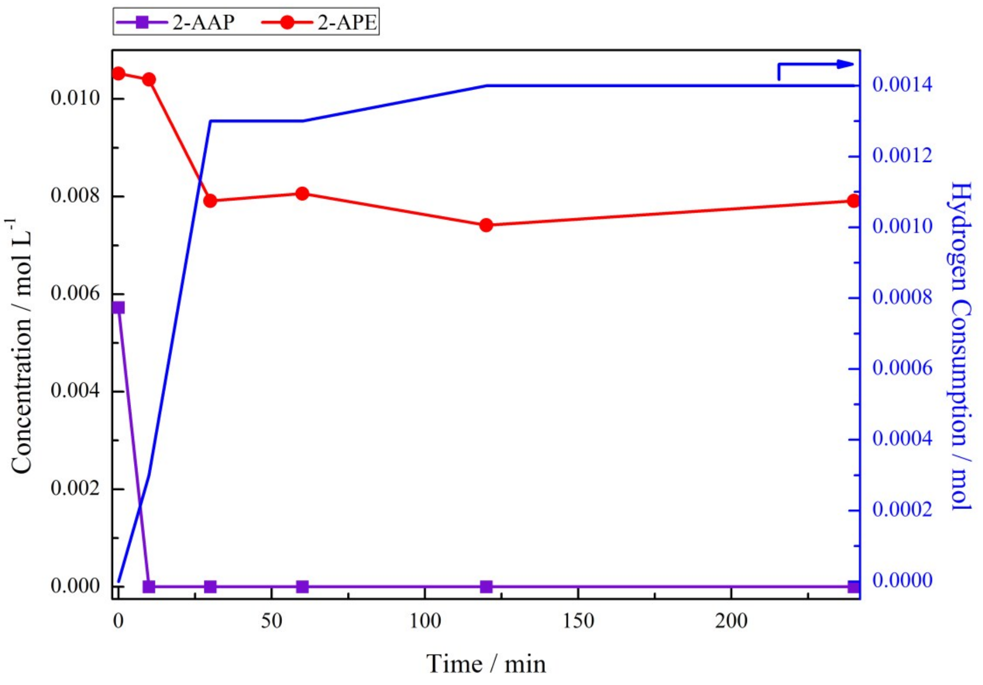

Figure S12. *Reaction profile and hydrogen uptake curve for the hydrogenation of 2-aminoacetophenone over 300 mg 5% Pd/C catalyst, ca. 2.75 mmol 2-aminoacetophenone in the absence of an acid as an auxiliary agent. Reaction carried out at 40 °C, 6 barg hydrogen with an agitation rate of 1050 r.p.m.. [2-APE = 2-amino-1-phenylethanol; PEA = phenethylamine].*

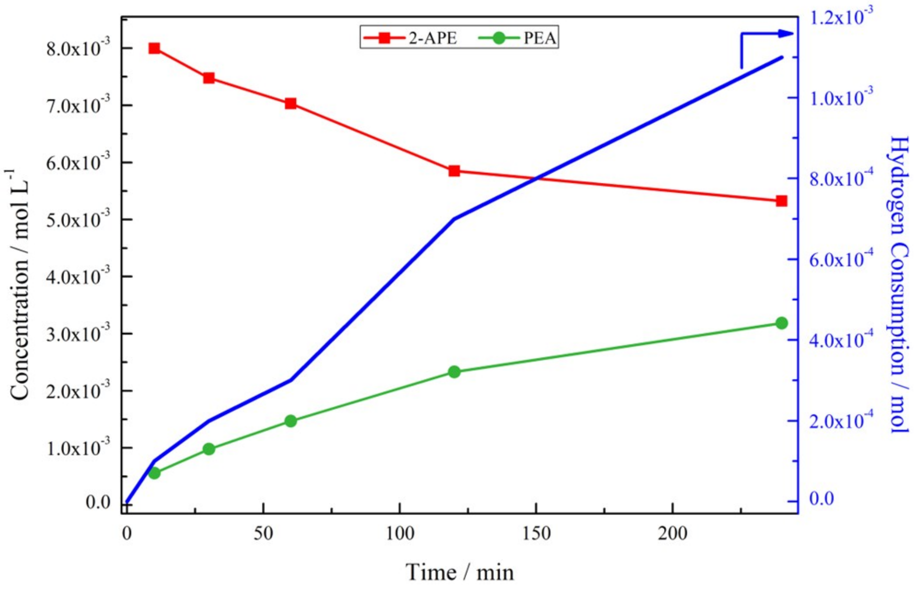

Figure S13. *Product profile and hydrogen uptake curve for the hydrogenation of 2-aminoacetophenonel over 300 mg 5% Pd/C catalyst, ca. 2.75 mmol 2-aminoacetophenone with 2 molar equivalents of* H_2SO_4*. Reaction carried out at 40 °C, 6 barg hydrogen with an agitation rate of 1050 r.p.m. [2-APE = 2-amino-1-phenylethanol; PEA = phenethylamine]. It should be noted that no data for 2-aminoacetophenenone was recorded due to the experimental set-up, which requires a set 'charging time' to allow the reaction pressure to be obtained. As such, and as a consequence of the rapid nature of the reaction, the starting material was completely consumed by the time the reaction was officially commenced.*

References

[a] S. Brunauer, L.S. Deming, W.E. Deming and E. Teller, *J. Am. Chem. Soc.*, 1940, **62**, 1723.

[b] K.S.W. Sing and R.T. Williams, *Adsorpt. Sci. Technol.*, 2004, **22**(10), 773.

[c] J. McGregor, Z. Huang, E.P.J. Parrott, J.A. Zeitler, K.L. Nguyen, J.M. Rawson, A. Carley, T.W. Hansen, J-P. Tessonnier, D.S. Su, D. Teschner, E.M. Vass, A. Knop-Gericker, R. Schlögl and L.F. Gladden, *J. Catal.*, 2010, **269**, 329.

[d] S. Rustler, V. Windeisen, A. Chmura, B.C.M. Fernandes, C. Kiziak and A. Stolz, *Enzy. Microb. Technol.*, 2007, **40**, 598.

[e] F. Pinna, F. Menegazzo, M. Signoretto. P. Canton, G. Fagherazzi and N. Pernicone, *Appl. Catal. A:Gen.*, 2001, **219**, 195.
